# Full Cycle Audit on Health Appointment Attendance: Comparative Analysis of Initial Audit and Reaudit Findings in a Psychiatric Care Setting

**DOI:** 10.1192/bjo.2024.638

**Published:** 2024-08-01

**Authors:** Ramy Teama, Keith Reid, Qudus Lawal

**Affiliations:** CNTW NHS TRUST, Newcastle upon Tyne, United Kingdom

## Abstract

**Aims:**

The full cycle audit aimed to evaluate and enhance attendance rates at health appointments in a psychiatric care setting. The initial audit (Phase 1) identified baseline attendance rates and underlying factors contributing to missed appointments. The reaudit (Phase 2) was conducted to assess the effectiveness of implemented interventions from Phase 1 and to identify areas for continued improvement.

**Methods:**

Both phases employed a retrospective evaluation methodology. Phase 1 reviewed records of 23 patients over two years, totaling 89 appointments. Phase 2, conducted as a follow-up, involved 19 patients with 39 appointments over a six-month period. Data collected included the number of attended and missed appointments, and reasons for non-attendance. Interventions after Phase 1 focused on addressing identified issues such as patient transfers, leave protocols, and transportation challenges.

**Results:**

Phase 1 recorded an attendance rate of 68.5%, with the missed appointment rate at 25.8%. Common reasons for non-attendance included patient decline and unclear reasons. Phase 2 showed a slight improvement in attendance rates (71.8%) but also an increased missed appointment rate (28.2%). Notable reasons for missed appointments in Phase 2 included patients on leave, ward cancellations, and transportation issues. The comparison revealed an improvement in attendance rates post-interventions, though challenges persisted, particularly in patient leaves and transportation.

The chi-square statistic is 2.2893 and the p-value is 0.3183. This indicates that there is no statistically significant difference between the attendance rates in Phase 1 and Phase 2. This suggests that the changes implemented between the two phases did not result in a statistically significant difference in attendance rates.

**Conclusion:**

The full cycle audit demonstrated marginal improvements in appointment attendance rates following targeted interventions. While Phase 2 showed a higher attendance rate, it also highlighted ongoing challenges, particularly in managing patient leaves and transportation. These findings underscore the need for continuous monitoring and adaptable strategies to further enhance attendance rates. Recommendations include improved communication during patient transfers, proactive leave management, addressing transportation issues, and ongoing evaluation to sustain improvements in health appointment attendance in psychiatric settings.

